# First assessment of population size, population dynamics, and carrying capacity of free-roaming dogs in Karaj, Iran

**DOI:** 10.1371/journal.pone.0358766

**Published:** 2026-11-13

**Authors:** Amin Hekmat, Asghar Abdoli, Faraham Ahmadzadeh

**Affiliations:** Department of Biodiversity and Ecosystem Management, Environmental Sciences Research Institute, Shahid Beheshti University G. C, Tehran, Iran; GCS Medical College, INDIA

## Abstract

Dogs (*Canis familiaris*) were the first animals domesticated by humans, likely in Eurasia between 20,000–40,000 years ago, and remain deeply integrated into human societies. However, in many countries, free-roaming dog populations have expanded due to uncontrolled reproduction, abandonment, and widespread supplemental feeding. In Iran, scientific assessments of these populations are scarce despite growing ecological, social, and public health concerns. This study provides the first systematic estimation of population size, demographic structure, and environmental carrying capacity of free-roaming dogs in Karaj, Iran. We conducted six surveys across 35 randomly selected blocks between May and August 2024 using a modified WSPA block-survey method. Age class, sex, visible health status, and ear-tag information were collected. Vital demographic rates, including birth, death, survival, and growth, were calculated and incorporated into a Leslie matrix to model long-term population dynamics under sterilization and euthanasia scenarios. Carrying capacity was estimated using a model integrating human support, shelter availability, demographic structure, and ecological variables. The mean estimated population size was 7,459 dogs (95% CI ± 1,530). Human support was present in 94% of surveyed blocks, elevating the carrying capacity to 68,895 dogs. Projection models revealed that sterilization rates below 50% do not meaningfully reduce long-term population growth, whereas 75% sterilization leads to sustained decline. Euthanasia sharply reduces numbers but raises ethical concerns and produces demographic rejuvenation. This study establishes baseline demographic information for Karaj and highlights the key role of anthropogenic support in sustaining dog populations. These findings provide critical insights for developing evidence-based, humane, and sustainable dog population management policies.

## Introduction

The domestic dog (Canis familiaris) is widely recognized as the earliest animal domesticated by humans, with estimates of domestication ranging from 15,000 to over 100,000 years [1,2]. This long-standing association has allowed dogs to establish deep cultural, emotional, and ecological ties with humans. Their dependence on and proximity to human settlements enable them to surpass natural ecological constraints and expand globally alongside people, unlike most wildlife species [3–5]. However, irresponsible ownership, abandonment, and extensive supplemental feeding have led to a substantial rise in free-roaming dog populations worldwide, producing significant ecological, economic, and public health concerns [6,7]. Dogs, humans, and cats share more than 60 zoonotic pathogens [8,9], emphasizing the importance of effective management.

Previous studies highlight broad consequences of expanding free-roaming dog populations. Feldmann [10] identified early concerns regarding disease transmission, attacks, road accidents, and wildlife impacts. Brickner [11] detailed ecological disruptions in Israel, including predation, disease spread, and hybridization. Similar impacts were documented across North America [12], Brazil [13], Mongolia [14], and Europe [15]. These studies consistently underscore the need for science-based urban dog management.

In Iran, public media discussions on free-roaming dogs are largely emotional and often based on limited empirical evidence. They often rely on expert opinion rather than empirical data, contributing to differences in public perception regarding dog management. As a result, municipal dog management is commonly disrupted by public opposition, while scientific data on free-roaming dogs remain scarce. One of the few quantitative studies, conducted in Kerman (southeastern Iran), showed that without intervention, the free-roaming dog population would increase significantly over 5 and 10 years; however, 70% female sterilization could produce meaningful reductions [16]. Building on the necessity for data-driven management [17–19], this study addresses these knowledge gaps by (1) estimating the population size of free-roaming dogs in Karaj, (2) analyzing demographic structure and vital rates, (3) modeling long-term population dynamics using a Leslie matrix under multiple intervention scenarios and (4) estimating environmental carrying capacity by integrating ecological and anthropogenic variables. The results provide a foundation for evidence-based management across Iran and other countries with similar conditions.

## Materials and methods

### Study area

Karaj is the provincial capital of Alborz Province. It is located in 35° 65’ 45” to 35° 89’ 14” latitude and 50° 78’ 46” to 51° 04’ 62” longitude with 1250m of average altitude above sea level and 7.5% average slope. Karaj is the fourth most populated city in Iran and 22^nd^ metropolitan city of the Middle East. The area of the study region is 444.962 Km^2^ and the human population is approximately 1,956,267.

### Data collection

The study area was divided into 222 survey blocks using the World Society for the Protection of Animals (WSPA) method [20]. The WSPA block-count method is a survey technique in which the study area is divided into spatially defined blocks. A representative subset of blocks is then surveyed systematically, and observed counts are extrapolated to estimate the total population [20]. The base map of Iran (shapefile format) was obtained from the vector datasets provided by the National Cartographic Center of Iran (eshop.ncc.gov.ir). A 30-m spatial resolution Digital Elevation Model (DEM) was acquired from the ASTER Global Digital Elevation Model (ASTER GDEM) database (asterweb.jpl.nasa.gov) to derive the topographic characteristics of the study area. The urban layer for blocking was obtained from the spatial datasets provided by the municipality of the study area. All spatial datasets underwent quality control, preprocessing, standardization, and integration before being imported into QGIS for visualization. Each block was sized to allow full coverage within two hours using a slow-moving bicycle. Urban blocks with dense alleys were smaller, while suburban and barren land blocks were larger due to better visibility and ease of access. Blocks were randomly color-coded (green, blue, red, yellow), ensuring no adjacent blocks shared the same color. Red blocks were randomly selected for fieldwork, and 35 were ultimately chosen following WSPA recommendations [20] (Fig 1). By using the WSPA method, it is guaranteed that all of the study area, with all the different land uses, is included in the sampling process. The selected survey blocks were geographically distributed across the entire study area and included residential, commercial, industrial, suburban, and undeveloped zones. This design was intended to capture spatial heterogeneity in dog distribution and human activity patterns throughout Karaj and improve the representativeness of city-wide population estimates.

**Fig 1:**
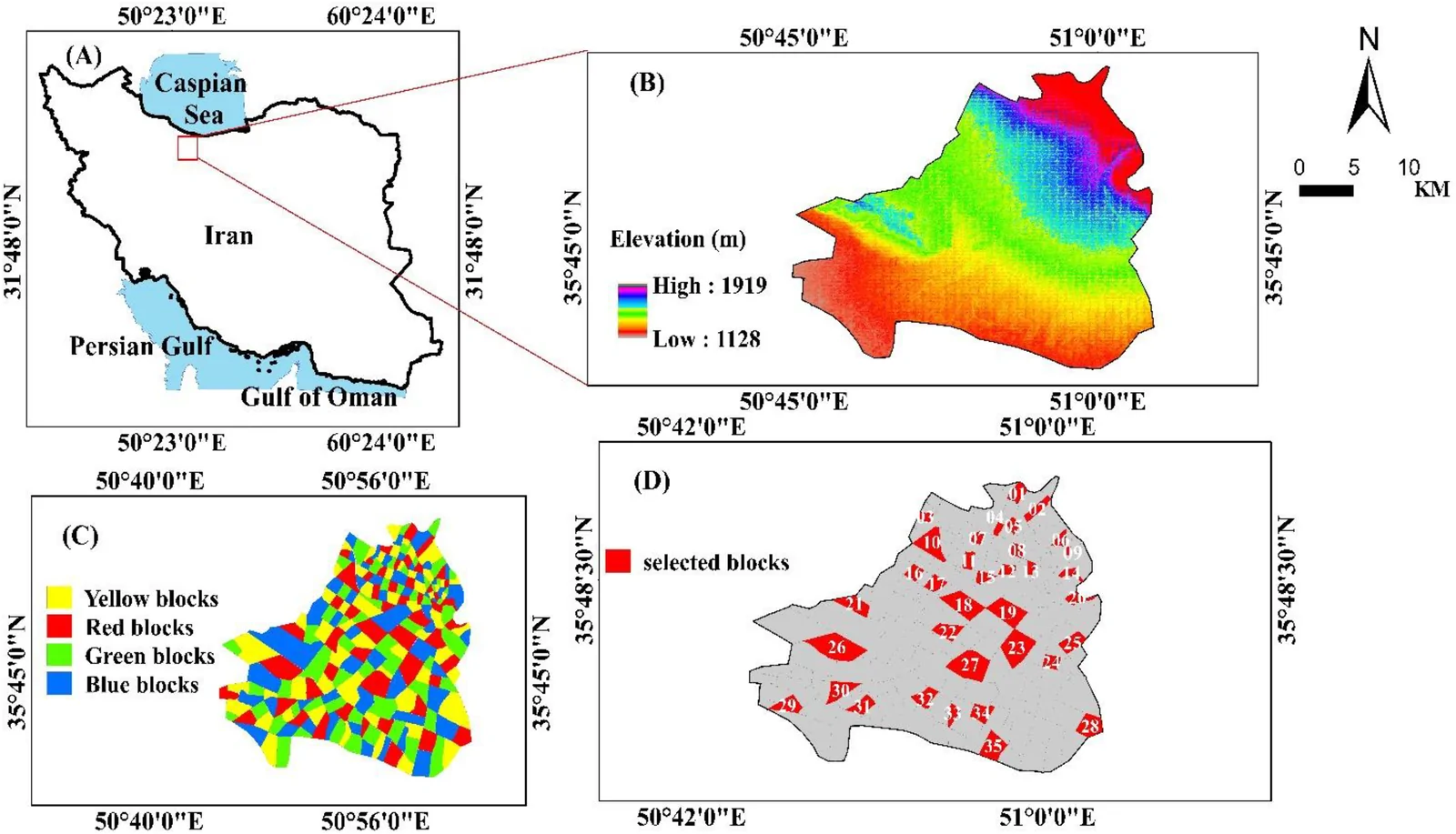
Location of Karaj city (the study area) and process of block selection using the WSPA method, red blocks were selected for sampling and the numbered blocks (shown at the bottom right corner) were the ultimate blocks in which the field surveys have been conducted (The base map of Iran (shapefile format) was obtained from the vector datasets provided by the National Cartographic Center of Iran (eshop.ncc.gov.ir). A 30-m spatial resolution Digital Elevation Model (DEM) was acquired from the ASTER Global Digital Elevation Model (ASTER GDEM) database (asterweb.jpl.nasa.gov) to derive the topographic characteristics of the study area. The urban layer for blocking was obtained from the spatial datasets provided by the municipality of the study area).

Field surveys were conducted between May and August 2024. Urban blocks were surveyed using bicycles, while off-road vehicles were used in rough suburban terrain. Binoculars aided accuracy, especially in suburban areas. Contrary to WSPA’s one-time survey recommendation, six rounds of surveys were conducted, three in the daytime (10:00–14:00) and three at night (22:00–02:00), to ensure data reliability for estimating vital rates [21,22]. Two blocks were surveyed per session, with immediate data entry into Excel.

Each survey recorded the following: total dog count; number of males and females; ear-tagged vs. non-tagged dogs; visible health status (ill dogs showing signs like limping or wounds); age classification (puppies: < 4 months, juveniles: 4–12 months, young adults: 1–7 years, older adults: > 7 years) based on size, skin, and dental features [23–25]; presence of active humans; and human support (e.g., feeding or signs of caregiving). For accurate age classification with dental features, a veterinarian was on the team during data collection sessions. For friendly dogs, dental examination was easy due to their friendly behavior and lack of fear of getting close to humans. But for non-friendly dogs or dogs fearful of humans (which wasn’t so many of them because of the constant feeding by local people), a piece of food was used as bait to make them get close. After they had eaten the bait and earned the slight trust of the fearful dogs, careful dental examinations were conducted to estimate the age. No dogs were captured or restrained. Although the WSPA blocking method automatically avoids recounting of dogs, after recording the information of each dog or pack of dogs, using smartphone, photographs were taken to make sure which of dogs are counted during every session.

### Data analysis

The data analysis started by estimating the total stray dog population in the study area using WSPA’s formula (Formula 1), applied separately to each of the six survey phases to identify population differences over time.


$$\begin{array}{c} \frac{Number\;of\;counted\;dogs}{\frac{number\;of\;sampled\;blocks}{total\;number\;of\;blocks}} \end{array}$$

Formula 1. Formula for estimating the number of stray dogs in the whole study area [20]

Standard deviations and confidence intervals (95% and 99%) were calculated and visualized. Also, vital rates (birth, survival, mortality, and growth) were calculated for each phase (Table 1). No formal statistical comparisons among demographic categories were conducted because the primary objective was population estimation and demographic characterization rather than hypothesis testing.

**Table 1:** Parameters, definition, and calculation methods of field survey data.

Parameter	Definition	Source/calculation method	Reference
N_n_	Population size at phase n	Field surveys	Field surveys
N_p_	Number of puppies	Field surveys	Field surveys
N_j_	Number of juveniles	Field surveys	Field surveys
N_a_	Number of young adults	Field surveys	Field surveys
N_o_	Number of older adults	Field surveys	Field surveys
N_m_	Number of males	Field surveys	Field surveys
N_f_	Number of females	Field surveys	Field surveys
Growth rate	The change in the number of individuals over a specific period of time	$\frac{\left(N_{n+\text{1}}\right)-(N_{n})}{N_{n}}$	De Roos, 2008;
Birth rate	The number of individuals born in a population in a given amount of time	$\frac{N_{p+\text{1}}}{N_{n}}$	Princée, 2016
Survival rate	The probability of survival of an individual from one age to the next	$\frac{N_{n+\text{1}}}{N_{n}}$	Lebreton et al., 1993; Aubry et al., 2010

Due to time and budget limitations, direct data on migration and mortality were not collected. Instead, mortality rates were estimated indirectly by calculating the expected population at the end of each phase (Formula 2). This was done using the survival rate, population size at the start and end of each phase, and the number of puppies, following methods outlined in previous studies [26–29].


$$\begin{array}{c} Expected\;Population\;at\;End\;of\;Phase=(Population\;at\;Begining\;of\;Phase\times Survival\;Rate)+Number\;of\;Puppies \end{array}$$

Formula 2. Calculation of the expected population at the end of each phase based on the survival rate

The expected population, calculated using survival and birth rates, was compared to the observed population to identify discrepancies, which suggest mortality or outward migration (Formulas 3 & 4) [30,31]. All vital rates for each phase were then derived and averaged across all six phases to establish consistent vital rates. These averages, along with dog and human population densities, were used to estimate the area's carrying capacity. This estimate provided the basis for developing management scenarios.


$$\begin{array}{c} Difference=Expected\;Population\;at\;End\;of\;Phase-Observed\;Population\;at\;End\;of\;Phase \end{array}$$

Formula 3. Difference between the expected population and the actual population at the end of each phase


$$\begin{array}{c} Death\;Rate=\frac{Difference}{Population\;at\;Begining\;of\;Phase} \end{array}$$

Formula 4. Calculation of the death rate

Carrying capacity (K) is defined as the maximum number of individuals an environment can sustain without ecological harm [32]. For free-roaming dogs, K is shaped by factors such as food and shelter availability, disease, human intervention, and social dynamics [13,30,33–36]. In urban areas, human support can elevate carrying capacity beyond natural limits [35,37,38]. This study estimated K using a formula incorporating ecological and human-related variables, aligned with established wildlife population management models (Formula 5) [39–43].


$$\begin{array}{c} K=\frac{A\times HSM\times SA\times HP\;to\;DP\times (\text{1}-Prop\;ill)\times ARF}{R} \end{array}$$

Formula 5. Calculation of carrying capacity for stray dogs in the study area.

In this formula, A denotes the area of the study region (km^2^). Human Support Mean (HSM) measures the average human support, such as feeding and sheltering, based on the proportion of sample blocks showing direct or indirect human support (33/35). Food containers (containing dog food or human food and food leftovers) left by humans for dogs were considered feeding. This is the proportion of blocks where support was observed [37,38,44,45]. Shelter Availability (SA) is calculated as the proportion of blocks with actual or potential shelter (31/35), which boosts survival and reproduction by offering protection. For this factor, human-built dog kennels were considered as actual shelters, and any structure or location capable of protecting from weather, human disturbance, or predators, including abandoned buildings, unfinished construction sites, drainage channels, dense vegetation patches, vacant lots containing debris, and other structures providing cover and protection, was counted as a potential shelter. During the field surveys, in several cases, dogs refuging in these potential shelters have been recorded; therefore, all of these cases are considered as shelter availability [3,46,47]. The HP to DP (Human Population to (estimated) Dog Population) Ratio compares the human population to the estimated stray dog population (1,956,267 / 7,459), reflecting the effect of human density on resource availability. Proportion of Ill Dogs (prop_ill) reduces carrying capacity by increasing mortality and decreasing reproductive potential [48–50]. Lastly, the Age Resource Factor adjusts for varying resource demands across age classes which was based on ecological theory and published studies, such as the higher needs of puppies and elderly dogs, by multiplying each age group's proportion by its Resource Demand Factor (RDF), as calculated in Formula 6.


$$\begin{array}{c} RDF=(prop\;puppy\times \text{1}.\text{5})+(prop\;juvenile\times \text{1}.\text{2})+(prop\;young\;adult\times \text{1})+(prop\;old\;adult\times \text{1}.\text{3}) \end{array}$$

Formula 6. Calculation of Resource Demand Factor for each age class

Where: Puppies (RDF = 1.5), Juveniles (RDF = 1.2), Young adults (RDF = 1), Old adults (RDF = 1.3). This formula reflects the varying levels of resource consumption and survival needs across different age groups [51–55].

The normalization factor (R) refines the carrying capacity estimate by incorporating key dynamic elements such as human activity, disease prevalence, and age structure. This factor ensures the model remains responsive to fluctuations in these variables, allowing for a more accurate reflection of the population's ecological limits [56–59]. It is calculated using the following formula (formula 7):


$$\begin{array}{c} R=\left(\text{1}+(\text{1}-AHPR)\right)\times (\text{1}+prop\;ill)\times (\text{1}+(yong\;adult\;female\;prop\times birth\;rate))\times (\text{1}+DPDR) \end{array}$$

Formula 7. Calculation of the normalization factor for carrying capacity

The normalization factor incorporates several key variables: Active Human Presence Rate (AHPR), representing human interaction like feeding and sheltering, which increases carrying capacity and were calculated by dividing blocks with active human presence into all blocks [5,35,60–62]; Proportion of ill dogs, which negatively impacts population resilience and carrying capacity due to higher mortality [63,64]; Young Adult Female Proportion, a vital factor for estimating reproductive capacity and population growth [65,66]; and the Dog-to-Human Population Density Ratio (DP to HP), which indicates competition for resources, with higher ratios linked to lower carrying capacity [3].

Population projections were conducted using a modified Leslie matrix model in R (version 4.4.2), allowing dynamic adjustment of birth and survival rates under different intervention scenarios. The Leslie matrix is an age-structured population projection model that estimates future population size by combining age-specific survival and reproductive rates. The method projects changes in population structure over time and is widely used for evaluating wildlife management scenarios and long-term demographic trends [67–70]. The model simulated population trends over 50 and 100 years across four scenarios: (1) no intervention, (2) 25% sterilization and euthanasia, (3) 50% intervention, and (4) 75% intervention. The model was calibrated with empirical data, including initial population size, carrying capacity, and vital rates, while intervention levels were theoretically defined.

## Results

The results of the raw counts recorded during the six survey phases are shown in Fig 2. As can be seen, there is a significant difference between the day phase (average of 1113 dogs) and night phase (average of 1239 dogs) raw counts.

**Fig 2:**
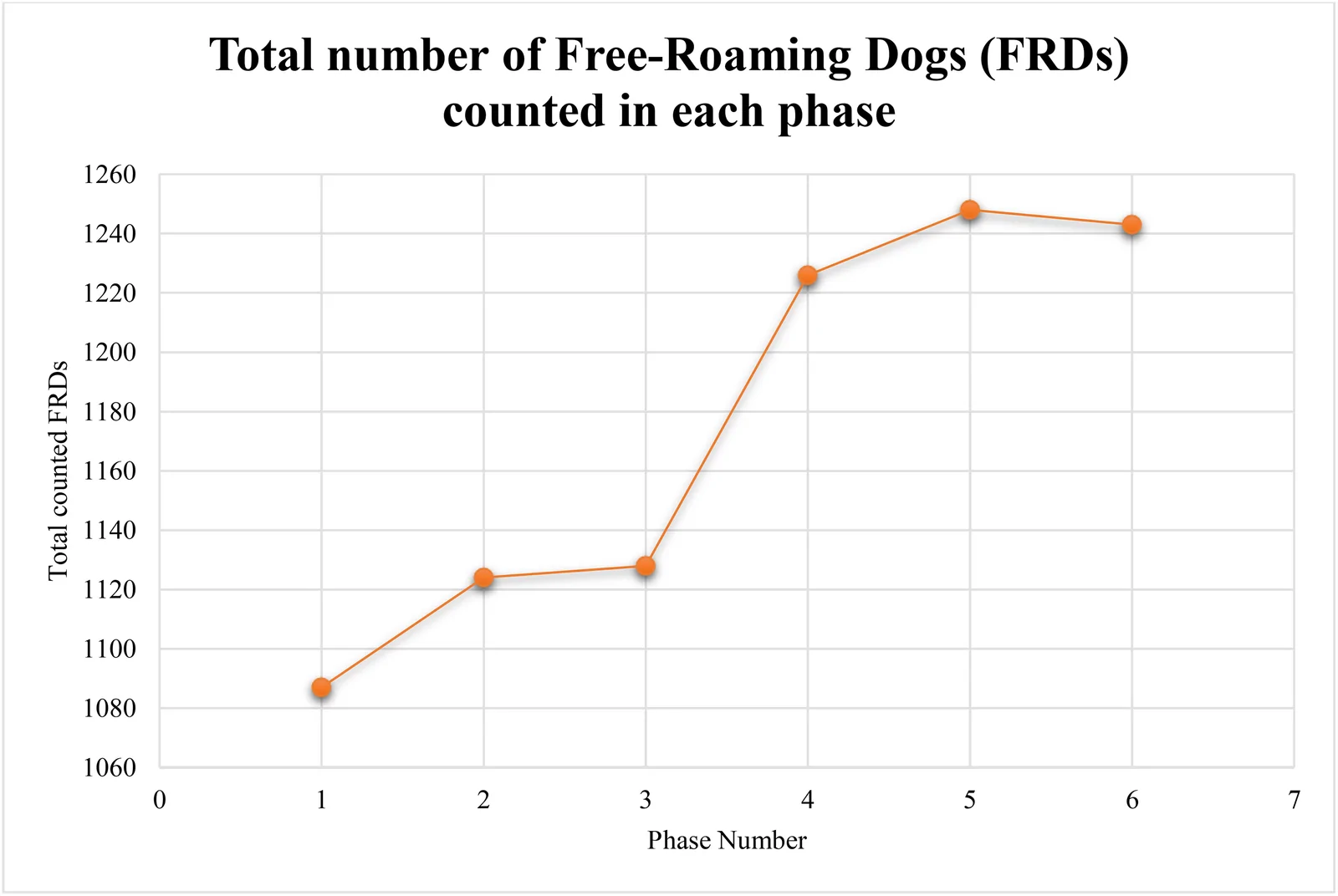
Raw counts of stray dogs in 6 phases in the study area.

Using these counts, the total number of stray dogs in the study area was estimated with the WSPA formula (Formula 1), yielding an average of 7,459 ± 1,530 dogs (95% CI) and 2,054 dogs (99% CI) in Karaj city (Fig 3). Additionally, the carrying capacity of the study area was calculated to be 68,895 dogs.

**Fig 3:**
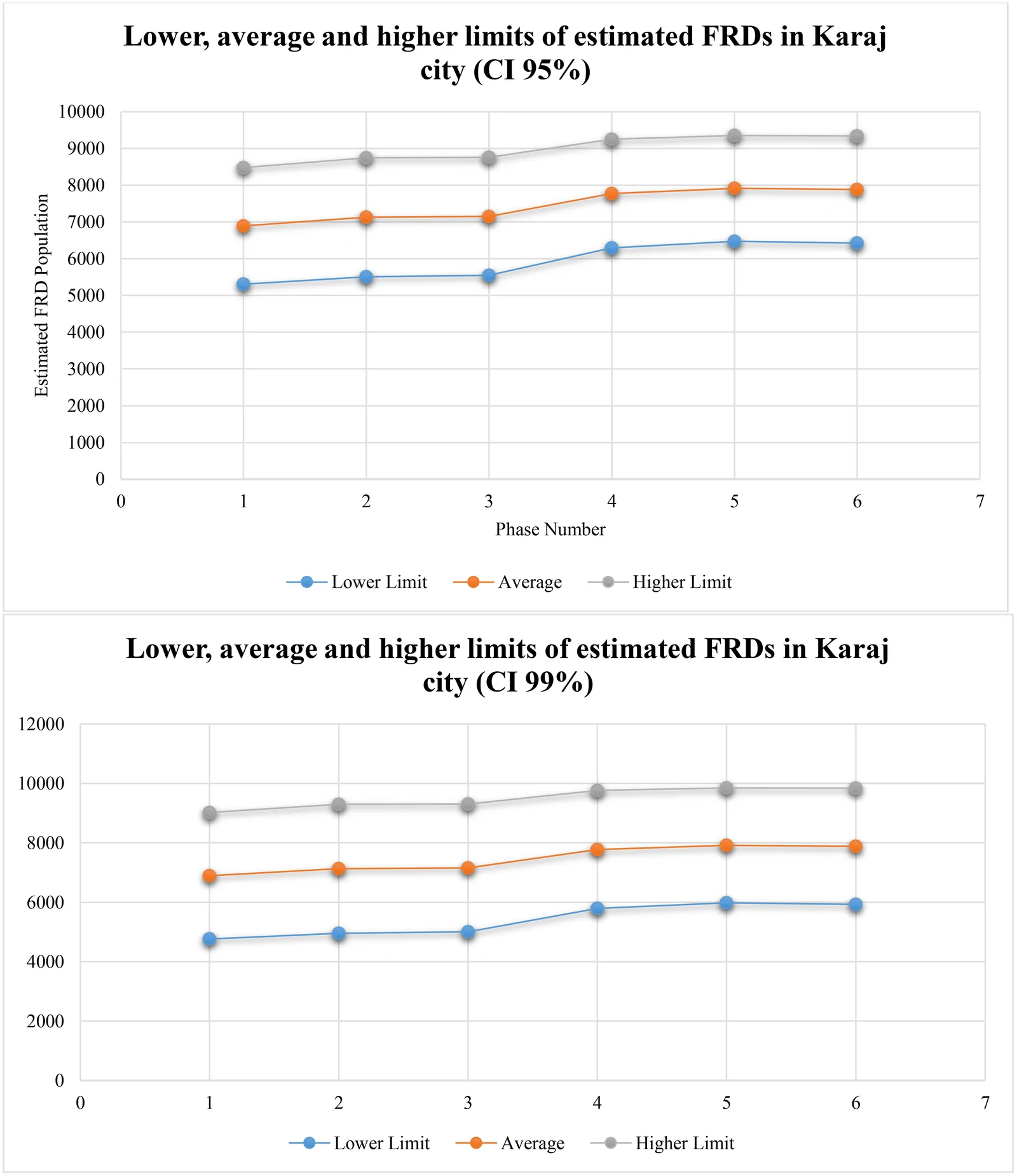
Estimated number of stray dogs with 95% and 99% confidence intervals using the WSPA method.

Across all surveys, females represented 37% of observations and males represented 63%. Young adults were the dominant age class, accounting for 70.5% of observed dogs, followed by juveniles (21%), older adults (5%), and puppies (3.5%). Ear-tagged individuals represented 17.5% of observed dogs, while visibly ill dogs accounted for 5%. Out of 35 surveyed blocks, 31 blocks had potential and actual shelters such as human-built dog kennels, abandoned buildings, unfinished construction sites, drainage channels, dense vegetation patches, and vacant lots containing debris. Also, 33 out of 35 surveyed blocks had evidence of human support, including food containers, deliberate feeding events, provision of water, improvised shelters, and repeated observations of dogs interacting with caregivers.

Using these data and the observed differences in stray dog numbers across phases, the average vital rates for population dynamics were estimated as follows: (a) average birth rate = 0.0708 (7.08%), (b) average death rate = 0.0398 (3.98%), (c) average growth rate = 0.0128 (1.28%), and (d) average survival rate = 0.9602 (96.02%). Based on these vital rates, long-term population projections over 50- and 100-year periods were conducted using the Leslie matrix approach (Fig 4). According to the results of the Leslie matrix approach and as seen in Fig 4, the population of Free-Roaming Dogs will reach the carrying capacity in 70 years. Furthermore, the results show that a 25% rate of sterilization proves to be ineffective in controlling the FRD population. In cases of 50% and 75% rates of sterilization, the results show that these rates are effective in controlling the FRD population, in which the 75% rate of sterilization is more effective. Sterilization at 50% could lower the population to half of the current size, whereas a 75% rate could lower the population near to zero in 100 years.

**Fig 4:**
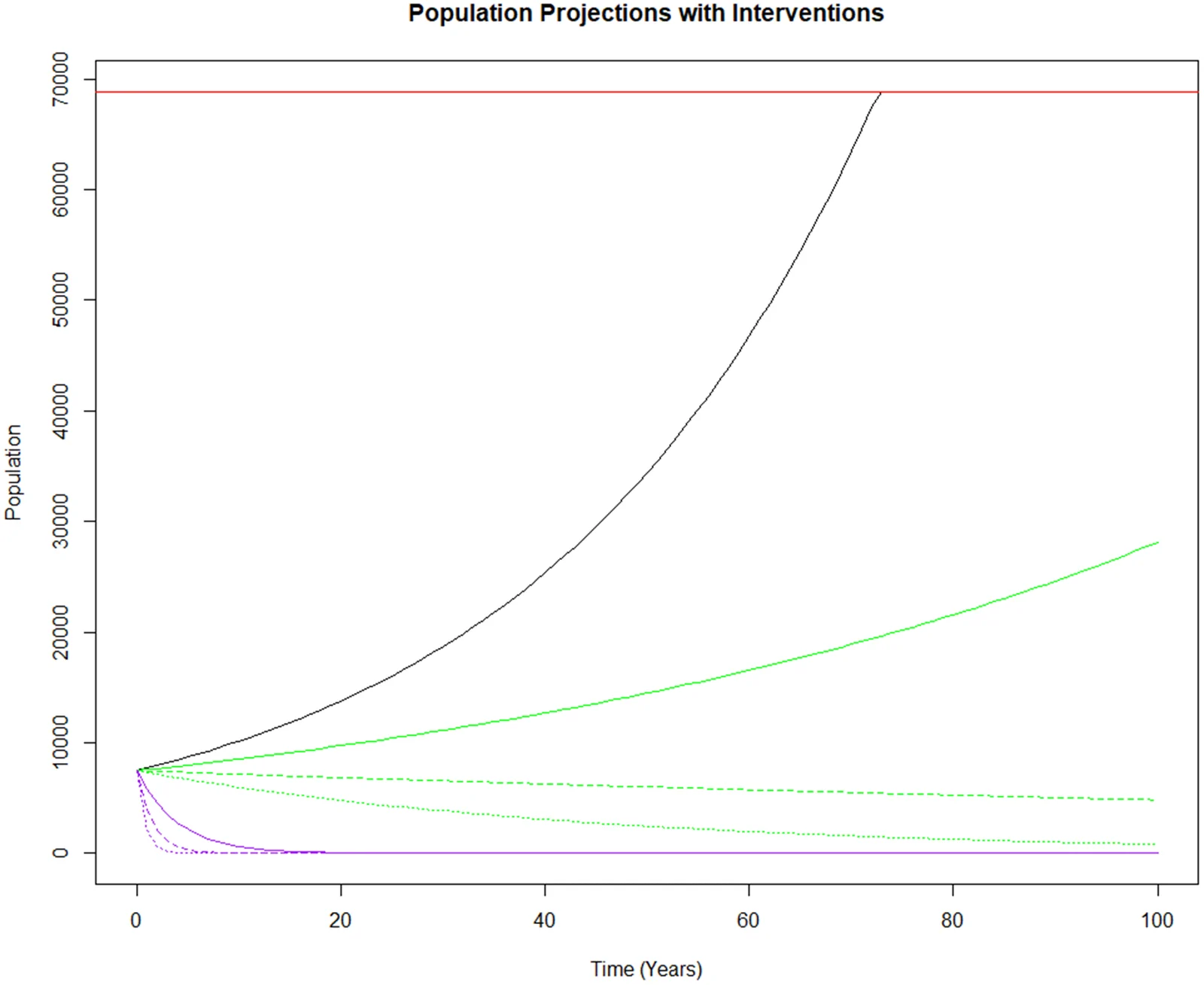
Projection of different scenarios of stray dog population changes over 100 years (green line: sterilization 25%, green dashed line: sterilization 50%, green dotted line: sterilization 75%. Purple line: euthanasia 25%, purple dashed line: euthanasia 50%, and purple dotted line: euthanasia 75%).

## Discussion

Field surveys revealed an average of 1,113 dogs during daytime phases and 1,239 during nighttime, with higher nighttime visibility likely due to the species’ nocturnal activity and avoidance of high human presence. Dogs were scarce or absent in blocks with high active human presence but reappeared at night (e.g., blocks 5, 9, 13). Blocks with high daytime human activity may reduce dog detectability because dogs often seek resting locations with lower disturbance levels and become more active during periods of reduced human activity. This behavioral adaptation has been reported in several urban dog studies supporting findings from Griss et al. [71], van der Laan et al. [72], and others. Puppies and juveniles were mainly recorded in blocks offering secluded shelters and minimal human traffic, highlighting the balance dogs seek between safety and their reliance on human-provided resources [5,62].

The WSPA-estimated population size averaged 7,459 ± 1,513 (95% CI). The estimated population size appears plausible given the extensive human support, widespread shelter availability, and relatively large urban area capable of sustaining substantial numbers of free-roaming dogs [16,73].

Karaj shows unusually high human support for stray dogs; 33 of 35 blocks showed signs of feeding, shelter, or treatment, even though few dogs are adopted. While well-intentioned, this support increases environmental carrying capacity, estimated at 68,895, which is almost ten times the current population [37,38,44,45]. Despite this, signs of overpopulation are already visible. The paradox lies in human support: abundant food and care reduce mortality, boost reproduction, and lessen competition, inflating the carrying capacity [43,66,74]. If such unregulated support continues, driven by public concern for animal welfare and limited awareness of long-term population consequences, the carrying capacity may rise further, posing risks to humans, wildlife, and ecosystems. These risks include dog attacks to human, thus increasing the physical damages plus the potential of increasing infections caused by dogs; hunting the urban animals; and in case of locations near natural areas, hunting wildlife species and inbreeding with other canids would affect genetic integrity of canids and natural ecosystems as well [75,76]. Current models predict the population will reach carrying capacity in 70 years, but continued support will shorten this timeline significantly [37,38,44,45].

Consistent with other studies, our models show that a 25% sterilization rate, regardless of sex, only slows population growth without reducing numbers [16]. A 50% rate leads to a moderate decline, while 75% sterilization reduces the population to below 3,000 over 50 years. Minor variations across studies are linked to environmental (including climate, food availability, habitat structure and shelter resources), cultural (public attitudes toward dogs and acceptance of feeding practices) and especially social (waste-management systems, responsible ownership, abandonment rates, municipal policies and community participation in control programs) factors, chiefly widespread supplementary feeding and community support. Even systematic sterilization becomes ineffective when citizens continue feeding and sheltering dogs excessively [62,77–81]. These actions inadvertently raise reproductive rates and undermine municipal control efforts.

In Iran, particularly in urban centers like Karaj, such behavior has severely impeded management. With no structured strategies in place and few attempts having faced challenges due to public concerns regarding animal welfare, social acceptance of management actions, and occasional legal or administrative objections, local authorities face increasing challenges. The effectiveness of management programs may be influenced by the level of public cooperation and community participation.

Modeling results show that euthanasia, even at a 25% rate, eliminates the dog population within 10 years; at 50% and 75%, this occurs in under 5 years. Despite its efficiency, euthanasia is not a sustainable or appropriate management strategy. Prior research highlights that while it may reduce the population quickly, it often leads to demographic rejuvenation [82]. In our study area, puppies and juveniles typically remain hidden near dens, while younger and older adults are more visible. Consequently, older adults, who are slower and easier to capture, are disproportionately removed, leading to a younger, more reproductive population over time [5,62,83,84]. Beyond ecological concerns, euthanasia is ethically controversial, challenges animal rights, and provokes strong public backlash, especially among community members concerned about animal welfare. Moreover, long-term studies show sterilization is more effective than euthanasia for population control. Therefore, euthanasia should be reserved only for critical situations such as 1. Animals with untreatable, severe illness or injury causing suffering 2. Confirmed rabies cases 3. Documented uncontrollable aggression posing immediate public safety threats. Confirmed rabies cases represent a serious public-health threat because rabies is nearly always fatal after symptom onset. International veterinary and public-health guidelines therefore permit humane euthanasia of infected animals to prevent transmission. Ultimately, euthanasia should never be used for population reduction alone [85].

The uncontrolled growth of stray dog populations, as reported in numerous global studies, leads to serious consequences for human and wildlife health, economic stability, and ecological balance, while also threatening the genetic integrity of wild canids [11,14,34,36,74,86,87]. Karaj is similarly affected, where increasing dog populations, with the added effect of human support without adequate awareness or education, may lead to a population explosion [33,88]. This continued supplementary feeding and resource provision may increase the environmental carrying capacity, allowing more dogs to survive [43,66,74]. Such growth not only results in visual pollution but also increases the risk of human-dog conflicts, especially affecting children [73,87,89]. In 2023, over 10,000 dog bite incidents were reported by the Ministry of Health in Alborz Province, and this statistic is expected to rise (it is noteworthy that even these statistics are not reliable due to a lack of continuous data collection by the government and/or insufficient accuracy). Without proper control and vaccination, disease and parasite transmission will escalate, causing greater public health and economic burdens [64,75,90,91]. Additionally, the growing dog population threatens urban animals such as free-ranging cats and wildlife species including jackals and foxes [8,64]. Traffic accidents involving dogs, along with associated casualties, economic losses, and pollution from carcasses, are also expected to rise [92,93]. Other issues caused by this phenomenon include surface water and soil contamination, visual pollution, and noise pollution from barking and inter-pack aggression [15].

Karaj is bordered by natural habitats in the north, northwest, southwest, and south, home to valuable wildlife such as wild goats (Capra aegagrus), red foxes (Vulpes vulpes), gray wolves (Canis lupus), golden jackals (Canis aureus), Eurasian lynx (Lynx lynx), hares, and rodents [94]. If the carrying capacity remains unchanged, a stray dog population explosion may lead to migration into nearby natural areas bordering sample blocks like 1, 2, 6, 14, 20, 3, 21, 26, and 29, due to the lack of shelter and resources in those areas [95–97]. In these environments, dogs will begin hunting, threatening wildlife populations, and may exhibit feral behavior due to the absence of human interaction [14]. Without annual vaccinations and veterinary care, disease and parasite transmission among dogs will rise, increasing the risk of cross-species infections through dog-wildlife interactions [14,98]. Moreover, Karaj’s surrounding mountainous and scenic areas, popular for recreation and ecotourism, could see rising dog attacks if stray dogs settle and become feral, posing serious risks to visitors [91,98].

Our modeling results suggest that high sterilization coverage (approximately 75%) may be effective for long-term population reduction under the conditions observed in Karaj. However, management decisions should also consider complementary approaches, including vaccination programs, public education, waste management, responsible ownership campaigns, abandonment prevention, and community engagement. Because demographic responses may vary among cities, management strategies should be adapted to local ecological and social conditions.

## Conclusion

This study as a part of first author’s Ph.D. thesis could be used as an efficient framework for any other cities in or outside Iran and although specific values may differ across cities, the methodological framework including block-based surveys, calculation of carrying capacity, photographic verification, and Leslie matrix projection is widely transferable to other urban environments in Iran and globally specifically in countries in which the issue of free-roaming dogs are not studied or the information on that matter is scarce (e.g., some of middle eastern countries).
