# Supplementary material for: First assessment of population size, population dynamics, and carrying capacity of free-roaming dogs in Karaj, Iran
Source: PLoS One. 2026 Nov 13;21(11):e0358766. doi: 10.1371/journal.pone.0358766 (PMC13766291; doi:10.1371/journal.pone.0358766)
Supplement: Supplementary file 1 [file pone.0358766.s002.docx]

**Dear Editor,**

We are grateful for being invited to submit a revised version of our manuscript " **First assessment of population size, population dynamics, and carrying capacity of free-roaming dogs in Karaj, Iran**". We note and appreciate the suggestions of the respected reviewers for improving the paper. We think that our revised manuscript has improved considerably due to their comments. On behalf of all co-authors, I am submitting the revised manuscript and our responses with details on how we addressed each reviewer’s comments.

Thank you again for considering our manuscript for publication in your journal. We believe that our original submission was suitable for the journal, and has now become an even better fit with the revision. I look forward to your decision soon regarding the outcome of the revised manuscript.

Sincerely.

6-26-2026

**Editor#:** Please include the following items when submitting your revised manuscript:

- A letter that responds to each point raised by the academic editor and reviewer(s). You should upload this letter as a separate file labeled 'Response to Reviewers'.
- A marked-up copy of your manuscript that highlights changes made to the original version. You should upload this as a separate file labeled 'Revised Manuscript with Track Changes'.
- An unmarked version of your revised paper without tracked changes. You should upload this as a separate file labeled 'Manuscript'.

When submitting your revision, we need you to address these additional requirements.

1. Please ensure that your manuscript meets PLOS ONE's style requirements, including those for file naming. The PLOS ONE style templates can be found at https://journals.plos.org/plosone/s/file?id=wjVg/PLOSOne_formatting_sample_main_body.pdf and https://journals.plos.org/plosone/s/file?id=ba62/PLOSOne_formatting_sample_title_authors_affiliations.pdf

2. Please note that PLOS One has specific guidelines on code sharing for submissions in which author-generated code underpins the findings in the manuscript. In these cases, we expect all author-generated code to be made available without restrictions upon publication of the work. Please review our guidelines at https://journals.plos.org/plosone/s/materials-and-software-sharing#loc-sharing-code and ensure that your code is shared in a way that follows best practice and facilitates reproducibility and reuse.

We note that you have provided funding information that is not currently declared in your Funding Statement. However, funding information should not appear in the Acknowledgments section or other areas of your manuscript. We will only publish funding information present in the Funding Statement section of the online submission form. Please remove any funding-related text from the manuscript and let us know how you would like to update your Funding Statement.

**RE:** We thank you for the opportunity to revise our manuscript, and especially thank the reviewers for their comprehensive reviews and constructive comments. We have thoroughly revised our manuscript based on those comments. A detailed description of how we addressed each comment is provided below. We have also included a track-change version of the manuscript, as well as a revised version without any tracked changes. We have edited the titles of figures and tables according to journals guidelines. Also, we have added the author-generated codes to our digital repository so it would be available for anyone and we have removed the Funding Declaration from the manuscript.

**Editor#:** Please include a separate caption for each figure in your manuscript

**RE:** Separate caption for each figure in manuscript has been added

**Editor#:** We note that Figure 1 in your submission contain map images which may be copyrighted. All PLOS content is published under the Creative Commons Attribution License (CC BY 4.0), which means that the manuscript, images, and Supporting Information files will be freely available online, and any third party is permitted to access, download, copy, distribute, and use these materials in any way, even commercially, with proper attribution. For these reasons, we cannot publish previously copyrighted maps or satellite images created using proprietary data, such as Google software (Google Maps, Street View, and Earth). For more information, see our copyright guidelines: <http://journals.plos.org/plosone/s/licenses-and-copyright>.

We require you to either (1) present written permission from the copyright holder to publish these figures specifically under the CC BY 4.0 license, or (2) remove the figures from your submission:

1. You may seek permission from the original copyright holder of Figure(s) [#] to publish the content specifically under the CC BY 4.0 license.

We recommend that you contact the original copyright holder with the Content Permission Form (<http://journals.plos.org/plosone/s/file?id=7c09/content-permission-form.pdf>) and the following text:

“I request permission for the open-access journal PLOS ONE to publish XXX under the Creative Commons Attribution License (CCAL) CC BY 4.0 (<http://creativecommons.org/licenses/by/4.0/>). Please be aware that this license allows unrestricted use and distribution, even commercially, by third parties. Please reply and provide explicit written permission to publish XXX under a CC BY license and complete the attached form.”

Please upload the completed Content Permission Form or other proof of granted permissions as an "Other" file with your submission.

In the figure caption of the copyrighted figure, please include the following text: “Reprinted from [ref] under a CC BY license, with permission from [name of publisher], original copyright [original copyright year].”

2. If you are unable to obtain permission from the original copyright holder to publish these figures under the CC BY 4.0 license or if the copyright holder’s requirements are incompatible with the CC BY 4.0 license, please either i) remove the figure or ii) supply a replacement figure that complies with the CC BY 4.0 license. Please check copyright information on all replacement figures and update the figure caption with source information. If applicable, please specify in the figure caption text when a figure is similar but not identical to the original image and is therefore for illustrative purposes only.

The following resources for replacing copyrighted map figures may be helpful:

USGS National Map Viewer (public domain): <http://viewer.nationalmap.gov/viewer/>

The Gateway to Astronaut Photography of Earth (public domain): <http://eol.jsc.nasa.gov/sseop/clickmap/>

Maps at the CIA (public domain): <https://www.cia.gov/library/publications/the-world-factbook/index.html> and <https://www.cia.gov/library/publications/cia-maps-publications/index.html>

NASA Earth Observatory (public domain): <http://earthobservatory.nasa.gov/>

Landsat: <http://landsat.visibleearth.nasa.gov/>

USGS EROS (Earth Resources Observatory and Science (EROS) Center) (public domain): [http://eros.usgs.gov/#](http://eros.usgs.gov/)

Natural Earth (public domain): <http://www.naturalearthdata.com/>

**RE:** We have deleted the map image from google earth and replaced it by another author-created map. The base map of Iran (shapefile format) was obtained from the vector datasets provided by the National Cartographic Center of Iran (eshop.ncc.gov.ir). A 30-m spatial resolution Digital Elevation Model (DEM) was acquired from the ASTER Global Digital Elevation Model (ASTER GDEM) database (asterweb.jpl.nasa.gov) to derive the topographic characteristics of the study area (lines 87-91). We also replaced and uploaded new maps and information into both “figures” file and our file inventory.

**Reviewer #1:** The manuscript would benefit from careful language editing to improve readability and consistency. Numerous minor grammatical issues and awkward expressions are present throughout the text. A few examples are listed below.

**RE:** Thank you for pointing out the typing errors of the manuscript. We have read them all and carefully edited and corrected the sentences.

**Reviewer #1:** Line 67 – “This study addresses…” should not be capitalized after the previous clause.

**RE:** Corrected.

**Reviewer #1:** Line 74 – “Material & Methods” should be written as “Materials and Methods.”

**RE:** Corrected.

**Reviewer #1:** Lines 76–79 – The study area description needs rewriting for clarity and grammatical accuracy. The coordinates and descriptive text are difficult to follow in the current form.

**RE:** Thank you for pointing out the difficulty and unclarity of the section. We have edited the study area section to something clear and easy to understand (lines 76-80).

**Reviewer #1:** Line 79 – “22nd metropolitan city of middle east” should read “22nd metropolitan city of the Middle East.”

**RE:** Corrected.

**Reviewer #1:** Line 80 – “Km2” should be written as “km².”

**RE:** Corrected.

**RE: Reviewer #1:** Line 87 – “color coded” should be written as “color-coded.”

**RE:** Corrected.

**Reviewer #1:** Line 89 – “check out WSPA, 2007” is informal and should be replaced with a standard citation format.

**RE:** It has been corrected and replaced with appropriate citation format (Line 93).

**Reviewer #1:** Line 104 – “veterinarian doctor” should simply read “veterinarian.”

**RE:** Corrected.

**Reviewer #1:** Line 105 – “dental analyze” should read “dental examination.”

**RE:** Corrected.

**Reviewer #1:** Line 107 – “human-scaring dogs” would be clearer as “dogs fearful of humans.”

**RE:** Corrected.

**Reviewer #1:** Line 111 – “smart cell phone” should be written as “smartphone.”

**RE:** Corrected.

**Reviewer #1:** Line 119 – “hole study area” should be corrected to “whole study area.”

**RE:** Corrected.

**Reviewer #1:** Line 207 – “The results of raw counting… have been shown” would read more clearly as “The raw counts recorded during the six survey phases are shown.”

**RE:** It has been corrected and replaced.

**Reviewer #1:** Line 289 – “statics are not reliable” should read “statistics are not reliable.”

**RE:** Corrected.

**Reviewer #1:** Line 367 – “Refferences” should be corrected to “References.”

**RE:** Corrected.

**Reviewer #1:** While the study provides useful field observations, several methodological aspects require clarification. The sampling design using the WSPA block method is mentioned, but it is not entirely clear how the selected blocks represent the entire city or how spatial variation across different urban zones was considered. Additional explanation of the sampling framework would help readers assess the representativeness of the estimates.

**RE:** We thank the reviewer for this valuable comment. Additional details have been added to the Data Collection section to clarify the sampling framework and representativeness of selected blocks (lines 83-86 and 94-99).

**Reviewer #1:** The calculation and interpretation of demographic parameters also require attention. For example, the reported survival value exceeding one is difficult to interpret biologically, since survival probabilities normally range between zero and one. This suggests that either the parameter has been defined differently or there may be an issue in the calculation. The authors should clearly explain how each demographic parameter was derived from the field data.

**RE:** We sincerely thank the reviewer for pointing out the miscalculation we had. We recalculated the vital rates and the survival rate is (correctly) calculated (lines 257-258). All the other vital rates were calculated correctly. Also, table 1 has more information with formulas on how each demographic parameter has been derived from the field survey.

**Reviewer #1:** The carrying capacity model presented in the manuscript is conceptually interesting, but the derivation of the formula and justification for parameter values are not sufficiently explained. Some variables appear to be based on assumptions rather than empirical measurements. Additional explanation of how the model was constructed and how sensitive the results are to parameter choices would strengthen this section.

**RE:** We thank the reviewer for this important observation. Additional text has been added describing the rationale for selecting model variables and acknowledging that some parameters were based on literature-supported assumptions rather than direct empirical measurements (line 195). We have cited the references for our assumptions as well (lines 202-204). Also, additional information about other parameters of the carrying capacity formula such as the shelter availability, human support (what direct observations or indicators counted as human support) etc. has been added (lines 176-177; 181-188; line 189 and line 195).

**Reviewer #1:** The discussion would also benefit from a more neutral scientific tone. In several places the manuscript refers to “emotional campaigns” or “uninformed supporters.” While the influence of public attitudes is an important aspect of dog population management, these statements would be better framed in a more neutral and analytical way.

**RE:** Thank you for this valuable advice. All of the statements mentioned by the reviewer has been corrected and replaced by more neutral words (e.g. lines 297-298; 322-326; 388; 355-356 etc.)

**Reviewer #1:** Finally, the management recommendations should be presented more cautiously. Policy recommendations such as sterilization and euthanasia strategies ideally require stronger empirical support or discussion of alternative management approaches, including vaccination programs, waste management, and community engagement.

**RE:** The concern of the reviewer for this comment is understandable and correct. We agree and have changed the section accordingly (lines 390-396).

**Reviewer #2:** The manuscript is quite interesting and also important to demonstrate the problem of free-roaming dog population increase and countries like Iran and similar areas around the world. However, the section of Results and Discussion need to be improved. There are results that have not been included in Results.

**RE:** We sincerely thank you for your time and consideration and your valuable insights and recommendations for improving our manuscript. We have read the manuscript carefully and edited the results and discussion sections according to your valuable comments.

**Reviewer #2:**

TITLE
- I do not think the title should start with this question "Is our city going to be ruled by dogs?". Authors should avoid this statement and reframe the title appropriately.

**RE:** It is a valuable point and we have changed the title to “**First assessment of population size, population dynamics, and carrying capacity of free-roaming dogs in Karaj, Iran**”.

**Reviewer #2:**

INTRODUCTION
- Line 67 – “This ….” Does not need the uppercase of “T”

**RE:** Corrected.

**Reviewer #2:**

Materials and Method
- Line 79 – “The area …” the word The needs the uppercase of “T”

**RE:** Corrected.

**Reviewer #2:** Line 82 – Write completely what is WSPA and then the acronym.

**RE:** Corrected and “World Society for the Protection of Animals” has been added (lines 82-83).

**Reviewer #2:** Line 90 – please briefly describe what is WSPA methodology for those who might not be aware of this.

**RE:** Thank you for your valuable comment, we have added the description for the WSPA method at lines 83-86.

**Reviewer #2:** Line 101 – Were the authors able to see the dental features of dogs while feeding them?

**RE:** Yes. After we used the bait to earn the trust fearful dogs (which wasn’t so many of them) careful dental examinations has been conducted. Additional explanations have also been added to the manuscript (lines 120-122).

**Reviewer #2:** Line 161-163 – it appears more as Discussion rather than Methodology.

**RE:** The unnecessary details have been deleted.

**Reviewer #2:** Line 163-164 – how did the authors define “potential shelter”? If one block has only one shelter, did the authors define that block as “shelter”? with only one in the entire block? Please clarify this point.

**RE:** This is a valuable point raised by the reviewer and additional explanations have been added to clarify the “shelter availability” and “potential shelters” at lines 181-188.

**Reviewer #2:** Line 166 – Define what is HP and DP. I guess it refers to human pop and dog pop.

**RE:** It is the human population to estimated dog population. We added the complete information for that variable to the manuscript (line 189).

**Reviewer #2:** Line 200 – Describe briefly the method of Leslie matrix, what does the method do? I am not familiarized with that technique and maybe some future audience may not know about it.

**RE:** Thank you for such a valuable recommendation, we added the description of Leslie matrix to the manuscript (lines 226-231).

**Reviewer #2:**

RESULTS
- Figure 1 – Add a few details as legends, for instance what blocks were selected?

**RE:** We added necessary details such as the details the reviewer recommended as legends to the Figure 1.

**Reviewer #2:** Figure 2 – Stand for FRD, I guess authors refer to free-roaming dogs, but they have to say it in the legend.

**RE:** We added the “Free-Roaming Dog” to the legend.

**Reviewer #2:** Line 208 – 209 – Describe briefly what is presented in Figure 2, highlight the most important message or trend.

**RE:** The most important message of the Figure 2 is the difference between the counts of day and night phases which we have added to the manuscript (lines 237-239).

**Reviewer #2:** Line 214-217 – the rates should have units, per hundreds, per thousands, etc?

**RE:** The units for rates have been added (lines 256-258).

**Reviewer #2:** Figure 3 – is this figure necessary? Is it necessary to show two confidence intervals?

**RE:** The reviewer has every right to ask this question, but we felt obligated to put both confidence intervals for more clarity of the study. At discretion, the editorial team can delete one of them.

**Reviewer #2:** Figure 4 – Do the authors need to graphs? 50 years is included in the projection of 100 years. Is it necessary to separate them?

**RE:** This is a very valid point raised by the reviewer and we have deleted the 50-years projection.

**Reviewer #2:** Authors must describe the most relevant findings in Figure 4 (line 217-219). It is not sufficient to refers Figure 4 for readers to realize what is presented. Describe the figure.

**RE:** The complete description for findings of the Figure 4 has been added to the manuscript (lines 260-267)

**Reviewer #2:** I do not see descriptive statistics (males, females, average age, range, etc).

**RE:** We thank the reviewer for this valuable recommendation; we have added descriptive statistics to the results section of the manuscript (lines 246-253).

**Reviewer #2:** Is there any difference between variable categories?

**RE:** No formal statistical comparisons among demographic categories were conducted because the primary objective was population estimation and demographic characterization.

**Reviewer #2:** Overall, the Results are too scarce though I suspect that there is more information to include. It needs to be improved.

**RE:** We thank the reviewer for the insights and recommendations and using those insights we have tried to improve the manuscript.

**Reviewer #2:**

DISCUSSION
- Line 221- 223 – Those are results that have not been shown in Results.

**RE:** We added the relevant results to the “Results” section of the manuscript (lines 237-239).

**Reviewer #2:** Line 224 – how human presence might affect the visibility (presence) of dogs? Based on the literature, authors might want to explain deeper that relationship.

**RE:** The explanation of the relationship between human presence and visibility of dogs have been added to the manuscript (lines 274-280).

**Reviewer #2:** Line 226 – Again, authors are presenting important results that have not been shown in Results chapter. Describe how are the shelters look like? Abandoned houses? Boxes? Trash? It would be better if there are some photos to illustrate the work.

**RE:** We have added complete details about shelters in “Materials and Methods” and “Results” section in order to clarify this issue (lines 181-188; 250-253).

**Reviewer #2:** Line 230 – how reliability of average relates to area, human support and resources? Explain this idea.

**RE:** The estimated population size appears plausible given the extensive human support, widespread shelter availability, and relatively large urban area capable of sustaining substantial numbers of free-roaming dogs. We also added the explanation to the manuscript.

**Reviewer #2:** Line 232 – what are the “signs” of feeding, shelter or treatment? Give details. Actually, this information should be in Results and authors might try to interpret those results in Discussion.

**RE:** Valuable insight from the reviewer. The details about feeding and human support have been added to the “Results” section (lines 253-255). Also, additional information is available in the “Materials and Methods” section as well.

**Reviewer #2:** Line 240 – what type of risks? Make it clear.

**RE:** Detailed explanation about the risks of population explosion of free-roaming dogs has been added to the manuscript (lines 301-305).

**Reviewer #2:** The first paragraph contains several different ideas. I suggest authors should split the paragraph into two or three main concepts and discuss them independently.

**RE:** We thank the reviewer for this valuable recommendation. We have edited the first paragraph and turned it to more consistent and clear paragraphs (lines 274-308)

**Reviewer #2:** Line 247 -what studies did the author refer to? Also, explain what are the environmental, cultural and social factor that might influence dog population.

**RE:** The factors influencing free roaming dog populations have been added to the manuscript and referred studies are cited at the end of the sentence (lines 313-321).

**Reviewer #2:** Line 331-333 – They are not necessary to be in the manuscript in such a way.

**RE:** We have deleted the unnecessary information.

**Reviewer #2:** Line 273 – could the authors explain why euthanasia is recommended in “confirmed rabies cases”?

**RE:** Confirmed rabies cases represent a serious public-health threat because rabies is nearly always fatal after symptom onset. International veterinary and public-health guidelines therefore permit humane euthanasia of infected animals to prevent transmission (lines 345-348).

**Reviewer #2:** Line 295-296 – are cats considered “urban wildlife species”?

**RE:** This was a typing error and we thank you for pointing it out. We edited the sentence (lines 369-370).

**Reviewer #2:** It needs to be improved.

**RE:** We sincerely thank you for your time and consideration. Using your valuable insights and recommendations, we have edited the manuscript so it would be better in all aspects.

**Reviewer #2:**

CONCLUSION
- Reduce it to something more concrete.

**RE:** We edited the “Conclusion” section accordingly (lines 407-413).
